# Two Cases of Nephritis Produced by over Doses of Balsam Copaiba

**Published:** 1849-07

**Authors:** David W. Yandell

**Affiliations:** Louisville, Kentucky


Art. II.—*Two Cases of Nephritis produced by over doses of Balsam Copaiba.* By David W. Yandell, M.D., of Louisville, Kentucky.

Case I.—Mr. R. McK., a native of Ireland, aged 33 years, of lymphatic temperament and delicate constitution, sober and temperate habits, presented himself at my office on the 6th of May, complaining of a severe pain in the small of his back. A few weeks before leaving Ireland (in the fall of 1848) he contracted a blenorrhagia, for which he had used, as he expressed it, almost every known remedy without any benefit, unless it was a change in the character of the inflammation from a very violent and acute to a more mild and subacute degree. The drug of which he used the largest quantity and continued for the greatest length of time, was balsam copaiba. This, he declared, he had taken in tablespoonful doses three times a day, for two weeks together, without any unpleasant symptoms or any amelioration of the disease. Despairing, finally, of curing himself, he consulted a quack, who ordered him to resume the copabia, commencing with drachm doses four times a day, gradually increasing the quantity till the stomach revolted. He had been pursuing this treatment about three weeks when I saw him. At this time he was taking four tablespoonsful of the balsam in the twenty-four hours. The blenorrhagic discharge had entirely ceased two days before, but for four days previous (the 1st of May,) he had been considerably annoyed by the appearance of pains in the lumbar region, which, at first vague and not very severe, had rapidly increased in intensity and extent until they became almost intolerable, radiating downwards from the last dorsal vertebra to the pelvis and right leg, as far down as the knee, being most severe immediately over the right kidney and along the course of the spermatic cord and testicle of the same side, which was considerably retracted. The urine wich was voided very frequently, was accompanied during its flow, by a sensation of burning in the urethra, was deeper colored than natural, occasionally mixed with blood, and affording, after it had been discharged, a very strong odor of the balsam.

In addition to these symptoms, the patient complained of very great debility and general lassitude, almost constant nausea, and general uneasiness; had a slight chill every evening towards bedtime, which was followed by fever that continued during the greater part of the night. His appetite was gone; thirst very great; bowels costive; expression of countenance anxious, and general appearance that of a man much too unwell to be going about.

Notwithstanding this train of symptoms was rather more intense than that which sometimes follows the protracted or excessive use of copaiba, I contented myself with forbidding its further use, and enjoining rest, diluent drinks, anodynes, a purgative, and a suspensory bandage, hoping that, in removing the suspected cause, I should succeed in curing the disease.

Four days afterwards, the patient returned with the symptoms materially the same as when I first saw him. Contrary to my directions, he left the city in a few hours after his second visit for Cannelton, Ia., where he had obtained a situation in the factories, and I have not since heard from him.

The history of case II is complete; the symptoms are more clearly marked, and its value consequently is much greater than that of the one above reported, the termination of which I have not been able to give.

Case II.—The subject of this was a gentleman, 25 years old, of good constitution, and general robust health, though of somewhat irregular habits, being occasionally addicted to excesses in drink, who contracted, about five years ago, a blennorrhagia from which he has never since been entirely free. In the treatment of this affection, which from its mildness had long since almost ceased to attract his attention, he had used a great quantity and almost incredible variety of medicines, and among others, very large doses of balsam copaiba. Finding, after two or three years of almost continual medication, that the blenorrhagic disease still evinced its existence by an occasional discharge, amounting generally to a single drop of opake mucus, observed only on getting up in the morning, or after indulgence in venery, or a glass too much of brandy, he despaired of ever being entirely freed from it and ceased taking medicine altogether. Things continued in this way until the 14th of the present month, when he determined to adopt a plan of treatment, although it had failed before after repeated

2 *

trials, which was suggested to him by one of his friends. This consisted in taking half ounce doses of balsam copaiba three times a day.

He found, greatly to his delight, that the first three doses of the copaiba seemed to cut short the disease, though it left him with a dull, heavy pain in the loins, extending to the left leg, spermatic cord, and testicle of the same side, frequent desire to urinate, and a scalding sensation during the act. He took no more copaiba, but resorted to hip baths on going to bed. His condition not improving, he called at my office on the evening of the 16th and desired me to prescribe for him. Trusting, as in the former case, very much to nature, I directed him to stop the baths, take diluents and a purgative, wear a suspensory bandage, apply a belladonna plaster over the seat of the greatest pain, use light diet, and rest in the recumbent posture, all of which, except the latter, he most strictly observed.

On the 17th, the patient was somewhat alarmed at the appearance of blood in the urine, and, at my earnest solicitation, promised to keep his room. Upon examining the kidneys by pressure through the walls of the abdomen, I discovered great tenderness over the organ of the left side. The spermatic cord and testicle of that side were also extremely tender and painful, and it appeared to me somewhat tumefied. These latter symptoms belonging rather to epidydimitis than to nephritis, I began to suspect that the case might be of this nature, and accordingly, the patient being unwilling to submit to any other treatment, resorted to local applications to the parts, in the form of poultices, a lotion of equal proportions of laudanum and belladonna, diet, and elevation of the testicle.

18th. Patient passed a restless night; tongue coated; mouth dry; skin hot; pulse quick; urine, passed at short intervals, high colored and mixed with blood; pain and tenderness undiminished; considerable nausea. Notwithstanding my representations to the patient of the gravity of his disorder, I could not induce him to allow me to practice venesection, which, under the circumstances, I deemed advisable. Nor would he listen to the application of leeches, or the administration of emetic tartar, but, after much persuasion consented to being cupped, which operation was performed very extensively over the region of the kidneys. The belladonna and laudanum were continued, and cloths dipped in the solution were applied over the scarifications. He took, also, until his bowels were freely moved, a tablespoonful, every two hours, of the following mixture:

℞. Magnes. ʒiss.
Gum acacia,
Sacc. alb. ää q.s.
Aq. ℥iv.
Pil. hydrarg. gr.x.
Ant. et potass tartrat. gr.iv.
Tinct. rhei, ℥ij.
Ol. anis. gtt. iv.

In addition to the above, I prescribed half a tablespoonful, four times a day, of

℞. Infus. digital., ℥iv.

Tinct. digital., ʒj.

Tinct. opii, gtt. x.

M.

19th. Passed a more comfortable night; general appearance much improved; called up once to stool, complained of having experienced a sense of chilliness, followed by fever in the early part of the night; tongue natural; skin moist; less pain and tenderness; urine voided less frequently, no longer mixed with blood, but still high colored. Ordered frictions over the internal surface of the left thigh, testicle, and spermatic cord with equal parts of unguent. hydrarg. fort. and extr. belladon.; belladon. and laud., and digital. mixture continued as before. Ten grains of quin. sulph., pulv. ipecac. c., and five grains of hydrarg. submur. ordered for 5 P.M.; diet and diluent drinks.

20th. Patient had no fever, as on previous evening; slept well till towards day, when he was twice up to stool; tongue clean; some appetite; kidneys much less active; urine less high colored, indeed almost natural; pain subsided; still some tenderness. Quinine, calomel, and Dover's powder omitted; the local applications continued, as was the diuretic mixture, though in diminished quantity; patient allowed tea and toast.

21st. Patient rested well; has a good appetite; tenderness, except on long continued pressure, entirely gone; secretion of urine and the urine itself natural. Ceased my visits.

Dr. Cullen, in his Nosology, gives the following definition of nephritis: "Pyrexia; pain in the region of the kidneys, often following the course of the ureter; frequent desire to pass urine, which is either limpid or colorless, or very red; vomiting; numbness of the leg; retraction or pain of the testicle of the same side."

M. Rayer, in his very valuable work entitled *Traité des Maladies des Reins*, recognizes four varieties of the disease, named according to the parts of the kidney implicated.

1. *Nephritis*, where the gland itself is inflamed.

2. *Pyelitis*, where the pelvis and calices are the parts inflamed.

3. *Perinephritis*, where the inflammation is in the investing membrane.

4. *Pyelonephritis*, where both the pelvis and the granular structure are involved.

While pure idiopathic nephritis is acknowledged, by most writers to be a disease of very rare occurrence, symptomatic nephritis is by no means uncommon. Its most usual exciting cause arises from the tubuli uriniferi, the pelvis, or canal of the ureter, being obstructed or dammed up by the presence of calculi, or calculous matter. "But it may arise from other causes, as from a blow, or a bruise, or strain, from severe exercise, long confinement in a recumbent posture, plethora, *acrid diuretics*, excess in the use of spirituous liquors, poisons, etc."

The most usual effects of copaiba, when given in too large quantities, are, we presume, diarrhea, certain eruptions upon the skin, vomiting, which is caused either by an insurmountable repugnance, or by gastric irritation; and it may even produce enteritis, several cases of which Ricord is in the habit of mentioning in his clinical lectures.

In the translation of the lectures of this eminent surgeon, by Victor de Meric, the following paragraph occurs: "Copaiba may likewise give rise to lumbar pains, which have so often been mistaken for symptoms depending on urethral mucitis. Patients placed under the influence of this substance are often attacked by a sharp pain in the lumbar region, which they compare to the reception of repeated blows. Do not fall into error on this head, and be careful not to set this down as blennorrhagic nephritis, as some have done. For if renal blennorrhagia were looked upon as the cause of these, it would turn out to be a very frequent complication of the disease; whereas, on the contrary, it is extremely rare. A proof of the correctness of the opinion which ascribes the lumbar pain to copaiba alone, may be obtained in a couple of days in most of the cases we have to treat. Let the drug be given up and all the uneasiness in the back will disappear, thereby dispelling the idea that it depended upon the extension of the blennorrhagia of the kidneys."

My own experience coincides with that of M. Ricord. In a number of cases which I have seen, many of the symptoms of nephritis during the administration of copaiba disappeared in the course of forty-eight hours after the drug was relinquinished, and in all these instances the lumbar pains began to diminish, sometimes before, and always after the lapse of twenty-four hours. I have now under treatment for blennorrhagia two young gentlemen, one of whom had diarrhea, the other vomiting, and both lumbar pains, developed during the use of copaiba. On its being given up, all these unpleasant symptoms subsided. In the two cases reported, these pains, and the other symptoms denoting high inflammation of some portion of the genito-urinary apparatus, if not of the kidneys themselves, not only did not subside, but absolutely grew worse after the copaiba had been discontinued, in one of them four days, and in the other three days; and in both recovery was favored by diluents, a purgative, diet, and anodynes. The cessation of the use of copaiba, in ordinary cases, is alone sufficient to allay in a short time the lumbar pains produced by its administration. In the two cases just related, notwithstanding it had been discontinued for a much greater length of time than usually suffices for their disappearance, the lumbar pains, with the other symptoms, continued steadily and rapidly to increase in intensity.

May 30, 1849.

